# Detection of Pregnancy by Ergot

**Published:** 1859-10

**Authors:** 


*Detection of Pregnancy by Ergot.*—MESSRS. EDITORS: I do not recollect of ever having seen the use of ergot recommended for the purpose of detecting pregnancy in its earlier stages. For many years I have been in the habit of administering small doses of this drug for this purpose, and in my hands it has seldom failed of furnishing the evidence sought. The specific action of the medicine is not felt by an unimpregnated womb, while the gravid uterus, I believe, almost invariably responds to its action by some uneasiness in the back, but more particularly by pain in the upper part of the thighs, sufficiently to enable you to diagnosticate the case with great certainty. I have in many doubtful cases trusted to this test, and have very seldom been disappointed in my diagnosis. I will only add that the ergot can be given with entire safety in sufficient quantity to accomplish the object sought.

Your ob't serv't, W. W. C.

*Middleboro', March* 29, 1859.

*Boston Med. and Surg. Jour.*

